# Early‐life exposure to high‐fat diet influences brain health in aging mice

**DOI:** 10.1111/acel.13040

**Published:** 2019-09-27

**Authors:** Antonio Di Meco, Domenico Praticò

**Affiliations:** ^1^ Alzheimer’s Center at Temple Lewis Katz School of Medicine Temple University Philadelphia PA USA

**Keywords:** brain aging, diet, tau pathology, synapse, memory

## Abstract

Epidemiological studies have suggested a link between exposure to environmental factors early in life and susceptibility to neurodegenerative diseases in adulthood. In the short term, maternal diet is important for the growth and development of the fetus; however, it may also have long‐term effects on the health status of the offspring. Here, we investigate the effect that maternal high‐fat diet during gestation has on brain health of the offspring later in life. B6129SF2/J dams were fed a high‐fat diet during the 3 weeks’ gestation, then switched to standard chow diet after delivery. Offspring were always fed regular diet for the entire study and assessed in learning, memory, and brain pathology when 18 months old. Compared with offspring from control mothers, the ones from mothers exposed to high‐fat diet had significant better performance in learning and memory tests, which associated with an amelioration of synaptic integrity. Additionally, they had a significant reduction in total tau, a decrease in its pathological conformational changes and lower levels of caspase‐3‐cleaved isoforms. Our findings demonstrate that in utero exposure to high‐fat diet plays a protective role for offspring brain health later in life. They support the novel hypothesis that targeted dietary intervention specifically restricted to the gestation period could be implemented as preventative strategy for the age‐dependent decline in brain health.

## 1 INTRODUCTION

Current literature suggests a strong relationship between different environmental factors and health as well as the development of disease states of an individual (Kivipelto, Mangialasche, & Ngandu, 2018; Colpani et al., 2018; Hwang & Nho 2019; Rippe 2019).

Interestingly, these studies have also shown that the interaction between the environment and genetic makeup of each subject during the earliest stages of life is very important for the susceptibility to many diseases later in life (Capra, Tezza, Mazzei, & Boner, 2013).

During gestation, a healthy maternal environment is essential for the fetus well‐being since the mother represents the only environment for the developing organism. To this end, it is well established that maternal nutrition and dietary lifestyle are of critical importance for a normal/physiological fetal growth and development. The gestation period is typically associated with higher nutritional requests secondary to a change in the mother body and the high metabolic demands of the fetus; hence, any deficits could have a significant impact for the growing organism inside the mother (Stein, Pierik, Verrips, Susser, & Lumey, 2009).

Exposure to a variety of environmental factors during gestation and the effect on the offspring later in life has been widely investigated, among them heavy metals exposure, pesticides, steroids, and diverse dietary lifestyles (Bandoli et al., 2017; Ling et al., 2018; Soomro et al., 2019; Raghow 2012). In general, dietary habits have important and direct biological consequences on the metabolism and physiology of any individual. However, this fact is exacerbated during gestation a condition in which growth and development of the new organism is strictly dependent on the maternal nourishment. Thus, any change in the dietary habits of the mother could result in long‐term effects on the offspring. While high‐fat (HF) diet has been widely investigated and usually associated with a negative effect for the subjects who are directly and chronically exposed to it (Ludwig, Willett, Volek, & Neuhouser, 2018), no data are available on the influence that this diet may have on the offspring later in life if administered only during the gestation period.

Here, we investigated the effect that maternal exposure to HF diet specifically during the gestation period has on brain health of wild‐type mice offspring later in life.

## 2 RESULTS

### 2.1 Effect of gestational high‐fat diet on body weight and glucose tolerance

**Figure 1:**
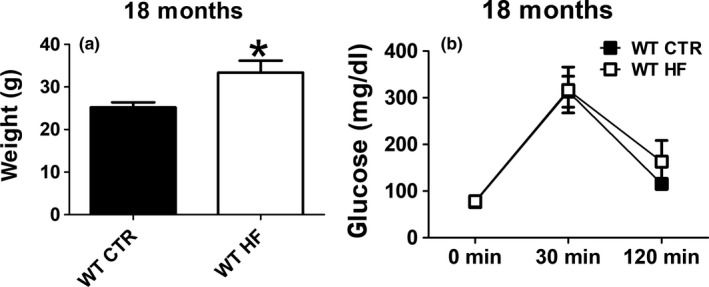
Effect of gestational high‐fat diet on body weight and blood glucose. (a) Body weight of wild‐type regular chow (WT CTR) and wild‐type high‐fat diet (WT HF) offspring at 18 months of age. (b) Glucose tolerance test of wild‐type regular chow (WT CTR) and wild‐type high‐fat diet (WT HF) offspring at 18 months of age (*p < .05)

Offspring from WT mothers fed either regular chow or high‐fat diet throughout gestation (WT regular chow: WT CTR; WT high‐fat diet: WT HF) were assessed for body weight at 18 months of age. WT HF offspring displayed a significant increase in body weight compared with WT CTR (Figure 1a). To investigate the effect of the gestational HF diet on blood glucose levels and insulin sensitivity in the offspring, mice were assessed in the glucose tolerance test at 18 months of age. As shown in Figure 1b, no differences were detected in the baseline blood glucose levels at fasting between the two groups. At the same time, no significant difference was detected in blood glucose levels at either 30 min or 120 min after glucose administration between the two groups (Figure 1b).

### 2.2 In utero exposure to high‐fat diet improves memory in aged mice

To assess cognitive function, offspring from the two groups were tested in several behavioral paradigms at 18 months of age. In particular, they were assessed in the Y‐maze test, fear conditioning, and Morris water maze (MWM) test.

**Figure 2:**
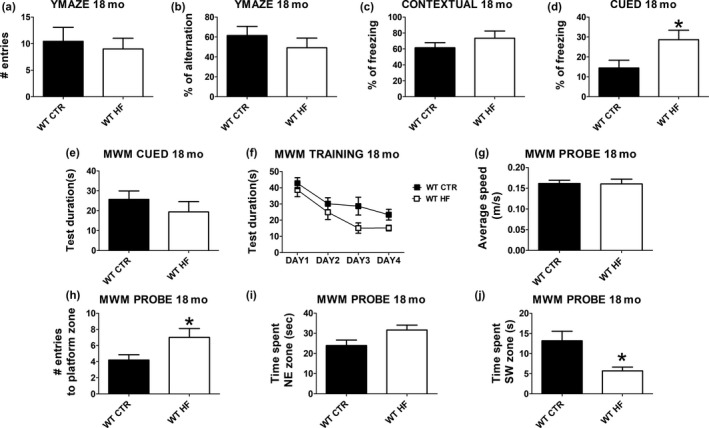
Effect of gestational high‐fat diet on cognitive function. (a) Number of entries and (b) percent alternation in the Y‐maze test for wild‐type regular chow (WT CTR) and wild‐type high‐fat diet (WT HF) offspring at 18 months of age. (c) Contextual and (b) cued phase of the fear conditioning test for wild‐type regular chow (WT CTR) and wild‐type high‐fat diet (WT HF) offspring at 18 months of age. (e) Cued, (f) training, and (g–j) probe phase in the Morris water maze test for wild‐type regular chow (WT CTR) and wild‐type high‐fat diet (WT HF) offspring at 18 months of age (*p < .05)

**Figure 3:**
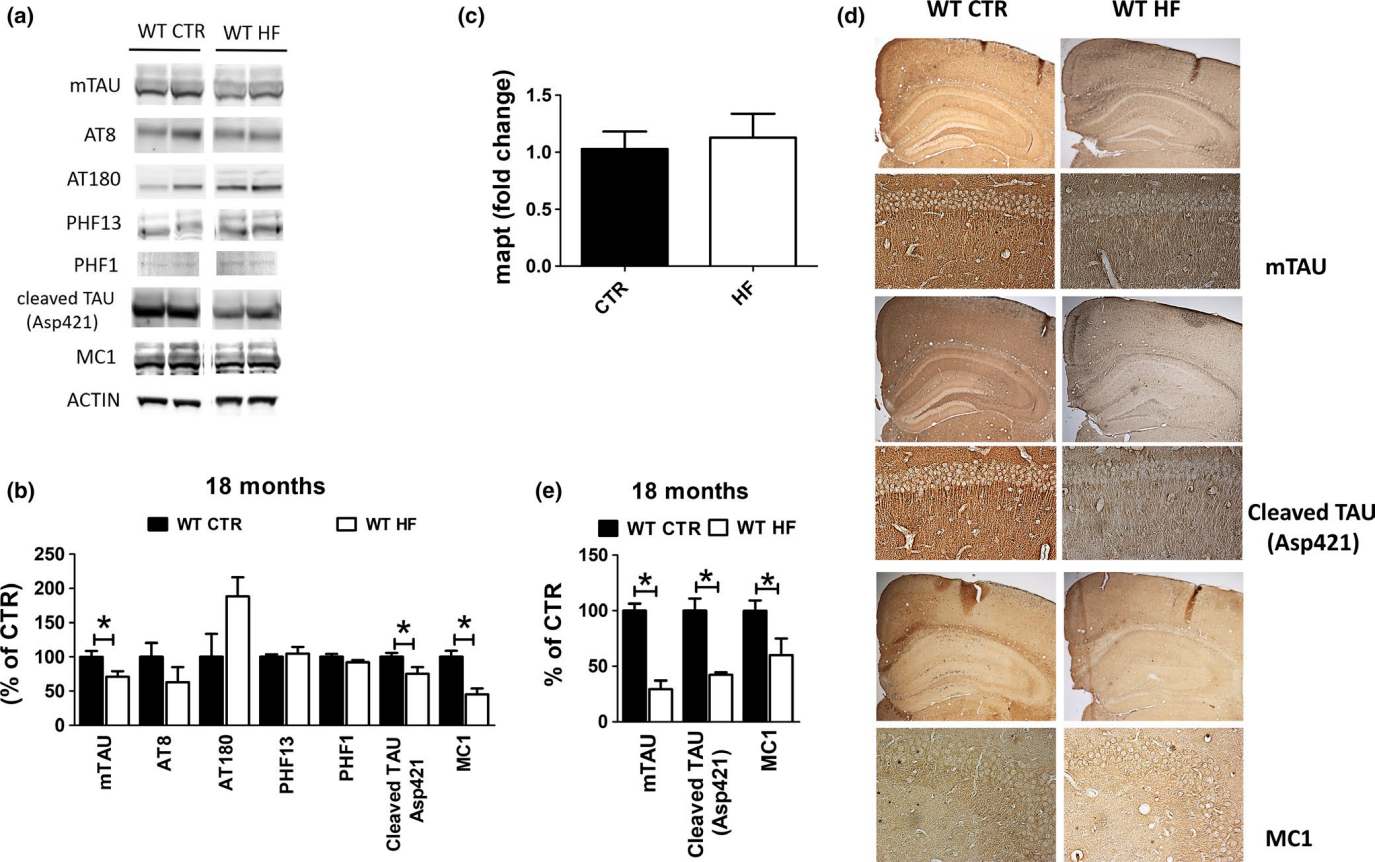
Effect of gestational high‐fat diet on tau brain pathology. (a) Protein levels of tau and its phosphorylated epitopes measured by Western blot in brain cortex of wild‐type regular chow (WT CTR) and wild‐type high‐fat diet (WT HF) offspring at 18 months of age. (b) mRNA levels of tau measured by qPCR in brain cortex of wild‐type regular chow (WT CTR) and wild‐type high‐fat diet (WT HF) offspring at 18 months of age. (c) Immunoreactivity for tau, caspase‐3‐cleaved tau, and aggregation prone tau (MC1) in brain sections of wild‐type regular chow (WT CTR) and wild‐type high‐fat diet (WT HF) offspring at 18 months of age. (d) Densitometry of immunoreactivities shown in panel a. (e) Quantitative analysis of the immunoreactivities shown in panel c. (*p < .05)

In the Y‐maze, which measure working memory, no differences were detected in the number of entries and percentage of alternation rate between CTR and HF offspring (Figure 2a,b). When challenged in the fear conditioning test, the HF offspring showed increased freezing behavior in the cued recall phase compared with control animals (Figure 2d). However, no difference was detected between the two groups in the contextual phase of the same test (Figure 2c). When assessed in the MWM paradigm, no differences were detected in the cued phase (Figure 2e) and average swimming speed (Figure 3g) of the MWM test when offspring from control mothers were compared with offspring from HF diet‐exposed mothers. In the training phase of the paradigm, the HF offspring were faster in learning the task of finding the platform when compared with offspring controls (Figure 2f). Moreover, the same offspring displayed significantly higher number of entries in the platform zone, a trend to increase for the time spent in the NW platform quadrant and a significant decrease for the time spent in the SW quadrant opposite to the platform (Figure 2h‐j).

### 2.3 Gestational high‐fat diet reduces tau pathology in the brain of aged mice

Next, we assessed total tau levels and its phosphorylation status in the brains of CTR and HF offspring at 18 months of age. As shown in Figure 3, HF offspring displayed a significant reduction in the protein levels of total tau compared with controls as measured by Western blot and immunohistochemistry. However, no changes were detected in the mRNA levels for tau in the same animals (Figure 3b). To better understand the effect of HF diet on tau brain metabolism, we measured several phosphorylated epitopes of tau, aggregation‐prone tau as recognized by MC1 antibody, and pathogenic tau cleaved at Asp421 in the same brain region of the two groups of animals. Although no significant changes in tau phosphorylation were observed between the two groups, HF offspring showed significantly lower aggregation‐prone (MC1 immunoreactivity) tau and tau cleaved at Asp421 levels when compared to CTR offspring as shown by both Western blot and immunohistochemistry (Figure 3a,c,d,e).

### 2.4 Gestational high‐fat diet reduces caspase‐3 activation in the brain of aged mice

**Figure 4:**
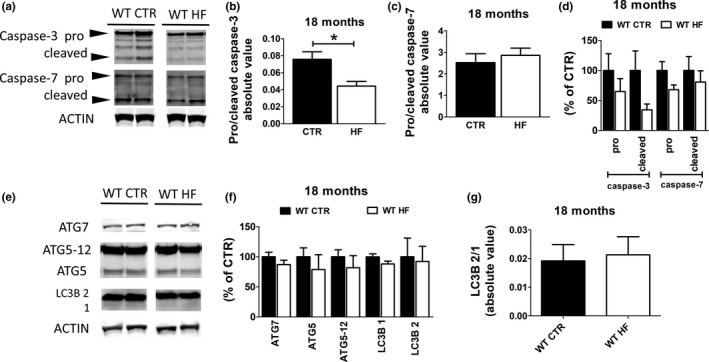
Effect of gestational high‐fat diet on tau clearance. (a) Protein levels of tau cleaving caspases measured by Western blot in brain cortex of wild‐type regular chow (WT CTR) and wild‐type high‐fat diet (WT HF) offspring at 18 months of age. (b–d) Densitometry of immunoreactivities shown in previous panel. (e) Protein levels of autophagy markers measured by Western blot in brain cortex of wild‐type regular chow (WT CTR) and wild‐type high‐fat diet (WT HF) offspring at 18 months of age. (f, g) Densitometry of immunoreactivities shown in the previous panel (*p < .05)

Since caspase‐3 cleaves tau at Asp421 priming it for aggregation into neurofibrillary tangles (Rissman et al., 2004), we assessed levels of caspase‐3, in the brain cortex of the two groups of animals. Indeed, 18‐month‐old WT HF offspring showed lower activation of capsase‐3 when compared to CTR offspring (Figure 4a–d). However, no changes were observed in the activation of another tau cleaving caspase, caspase‐7, in the brain of the same animals (Figure 4a–d). These findings suggest that gestational HF diet lowers tau aggregation in the offspring through modulation of its cleavage by caspase‐3.

Next, we assessed autophagy as another potential mechanism for tau clearance by measuring levels of some of its protein markers: ATG7, ATG5, ATG12, and LC3B1/2. As shown in Figure 4, no significant differences were detected in any of the autophagy markers considered in the brain of both groups of offspring (Figure 4 e‐g).

### 2.5 Gestational high‐fat diet ameliorates synaptic integrity in the brain of aged mice

**Figure 5:**
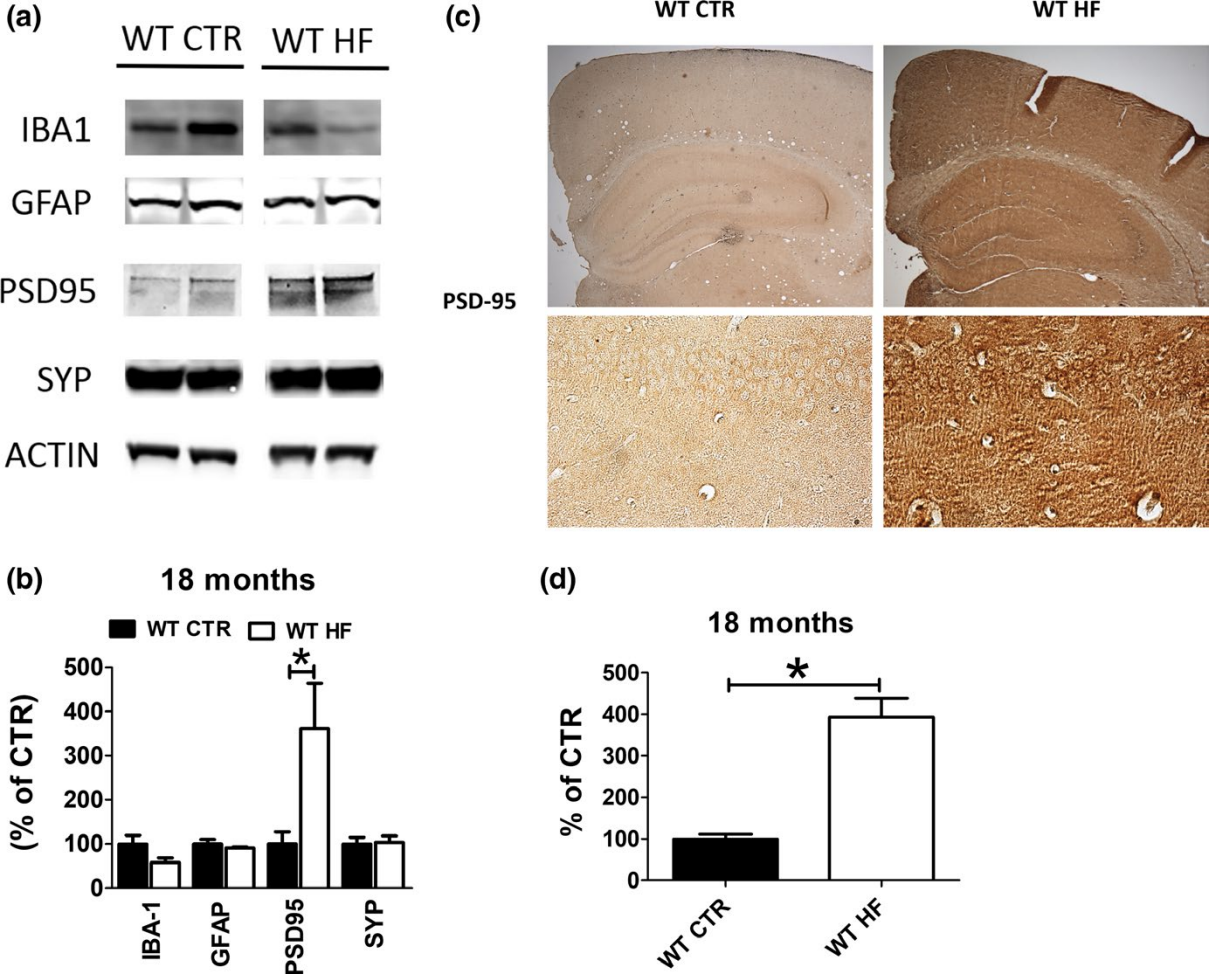
Effect of gestational high‐fat diet on synaptic integrity and neuroinflammation. (a) Protein levels of synaptic integrity and neuroinflammatory markers measured by Western blot in brain cortex of wild‐type regular chow (WT CTR) and wild‐type high‐fat diet (WT HF) offspring at 18 months of age. (b) Immunoreactivity for PSD‐95 in brain sections of wild‐type regular chow (WT CTR) and wild‐type high‐fat diet (WT HF) offspring at 18 months of age. (c) Densitometry of immunoreactivities shown in panel a. (d) Quantitative analysis of the immunoreactivities shown in panel b. (*p < .05)

Having observed an improvement of memory and learning abilities, next, we measured levels of pre‐ and post‐synaptic integrity markers synaptophysin (SYP) and postsynaptic density protein 95 (PSD‐95) in the brain of HF and CTR offspring. Although no change was observed for the steady‐state levels of SYP, a marked increase in PSD‐95 levels was observed in the brain of gestational HF diet offspring when compared with controls, indicating an improvement in overall synaptic integrity (Figure 5a,b).

Finally, neuroinflammatory markers such as glial fibrillary acidic protein (GFAP) and ionized calcium‐binding adapter molecule 1 (IBA1) were also measured in the brains of the two groups of offspring. As shown in Figure 5, no changes were observed in any of these cell neuroinflammatory markers between the two groups (Figure 5a,c).

## 3 DISCUSSION

In the present paper, we show that in utero exposure to HF diet has an effect on brain health in the offspring of WT mice later in life. In particular, we demonstrate that offspring from mothers exposed during the gestational period to this type of diet had better learning and memory functions, reduced pathological tau levels, and a significant improvement in synaptic integrity when compared to offspring from mothers on a standard diet.

Today, it is widely accepted that the early‐life phase for any living multicellular organism plays a crucial role not only for its development, but also for its general health (Capra et al., 2013). To this end, the literature has indicated that during these early stages the interactions between genes and external factors are important for the susceptibility to many pathological conditions later in life. This concept is now getting particular attention in the context of sporadic diseases, such as neurodegenerative disorders, for which in utero exposure to diverse factors has been investigated to better understand their complex pathophysiology. In general, the gestation period is characterized by a significant increase in the nutritional demands for the mother secondary to the fast‐growing nutritional need of the conceptus. Therefore, a diet rich in different elements, vitamins, and nutrients is the basis not only for healthy growth in utero but has long‐reaching effects for the health of the offspring later in life.

Considering that the period of intrauterine brain development is crucial for making connections among neurons and between neuronal and glial cells, it is not a surprise that evidence shows that exposure to environmental factors during gestation may have an effect on brain health. However, most of the studies conducted so far have focused on maternal HF diet consumption and subsequent obesity before pregnancy as important influencer of offspring behavior (Sullivan et al., 2015). Additionally, these investigations have often extended their experimental design and observation onto the early postnatal period during the lactation making difficult to differentiate between a pure in utero effect versus a postnatal exposure. The investigation of the precise timing of the exposure to the HF diet (e.g., gestation) is important since it could provide new insights into the mechanisms involved in the long‐term effects of maternal influence on the offspring brain health. With this goal in mind, we designed our study, which involved B6129SF2/J mice, as a model of normal aging, which were fed HF diet specifically during the gestation period. Then, we assessed the effect of this diet on memory, synaptic integrity, and tau neuropathology in the offspring at 18 months of age.

First, we evaluated whether gestational HF diet had any effect on the general metabolism by assessing body weight as well as glucose levels and insulin sensitivity. While we observed that compared with controls, offspring from HF mothers had a significant increase in total body weight, there were no significant differences between the two groups in basal glucose levels and glucose tolerance test, an in vivo measure of insulin sensitivity.

To assess their cognitive functions, offspring were administered a battery of behavioral tests, with the goal to assess different aspects of their learning and memory. Initially, we did not observe any significant difference between the two groups in the Y‐maze, a test that assesses working memory, since the percentage of alternations in the maze was undistinguishable when HF offspring were compared with controls. Importantly, we also did not see any difference in the number of entry into the different arms of the maze, a parameter that reflects the motor ability of the mice, suggesting that there were no differences in general motor activity between the two groups. Next, mice were assessed in the fear conditioning test, a measure of short‐term and long‐term associative memory. In this test, while we did not see any difference during the training phase, we observed that compared with controls offspring from HF mothers had a significant increase in the freezing time in the cue phase of the test, which reflects a better amygdala function. Interestingly, the same mice had also an improvement in the hippocampal‐dependent contextual phase, although the difference did not reach statistical significance (Sanders, Wiltgen, & Fanselow, 2003).

Finally, we tested the mice in the MWM paradigm, which is a measure of the hippocampal‐dependent reference memory and spatial learning. While there was no difference between the two groups in the cued test and the swimming speed, during the four days training compared with controls, mice from the HF mothers learned the task at a much faster rate. Additionally, in the probe test they had a significant increase in the number of entries into the platform zone and a significant reduction in the time spent in the opposite quadrant. Taken together, these data further confirm the improvement we observed in the fear conditioning task and provide a strong support for the beneficial effect of the gestational HF diet on the memory of WT aged mice. Our findings are in contrast with previous studies reporting a deleterious effect of maternal HF diet on learning and spatial memory (assessed by in the Morris water maze test) of the offspring (Martin, Jameson, Allan, & Lawrence, 2014). However, by contrast with our experimental design these studies implemented a more aggressive dietary approach treating dams during both gestation and throughout lactation. Moreover, the diet utilized had significantly higher caloric intake from fats (60%) compared to our milder approach (42%) (Martin et al., 2014). Also, the study was performed using 12–month‐old triple transgenic (3xTg) mouse model of Alzheimer's disease. We hypothesize that differences in timing of the exposure, age, and most importantly genetic background of the animals could also explain the discrepancy with our results.

Since we observed a beneficial effect on the behavior of the HF diet offspring, next we assessed whether this was associated with any modification at the synapse level. To this end, we measured pre‐ and a postsynaptic proteins, which are considered markers of synaptic integrity. Compared with offspring controls, we did not observe any changes in the steady‐state levels of synaptophysin, a presynaptic protein, in the HF diet offspring. By contrast, the latter group had a statistically significant increase in PSD‐95 levels, a well‐characterized postsynaptic protein marker.

Since aging is characterized by a progressive accumulation of phosphorylated tau protein and its conformational changes which together are considered precursors of the classical neurofibrillary tangles found in Alzheimer's disease (Sepulcre et al., 2017, 2016), next we investigated the effect of the HF diet on this aspect of brain aging. Compared with aged offspring controls, HF diet offspring had a significant reduction in the levels of total soluble tau protein, but the two groups were not different when phosphorylated tau isoforms were assayed. Interestingly, the changes in tau protein levels were not associated with changes in its mRNA, suggesting a post‐translational regulatory mechanism. However, we observed that brains from aged HF offspring had a significant reduction in the immunoreactivity for the antibody MC1, which recognizes pathological conformational changes of the tau protein (Jicha, Bowser, Kazam, & Davies, 1997). Additionally, compared with controls, offspring from the HF diet group had a significant decrease in the levels of tau cleaved at Asp421, which is known to be the product of caspase‐3 proteolytic activation in vivo, and implicated also in Alzheimer's disease pathogenesis (Rissman et al., 2004). Taken together, these data suggest that in utero exposure to HF diet‐dependent improvement in learning and memory is secondary to a significant reduction in the accumulation of tau pathological isoforms, which are known to directly modulate these aspects of the brain aging phenotype (Musi et al., 2018). Moreover, it seems that the beneficial effect of HF diet does not influence the levels of pathogenic phosphorylated isoforms of tau suggesting a primary effect on either tau clearance or tau proteolytic mechanisms. Our results are in contrast with previous reports demonstrating a deleterious effect of maternal HF diet on tau phosphorylation in the hippocampus (Martin et al., 2014). Similar to the behavioral findings, we believe that this discrepancy can be explained by the longer exposure of the dams to HF diet (gestation and lactation) and the higher caloric intake from fats (60%) implemented in that study.

In search for possible mechanisms responsible for the effect on tau clearance and accumulation, we first investigated whether the gestational HF diet had an effect on one of the major cellular system in charge of tau degradation and clearance: autophagy. To this end, we assayed some of the most common protein markers that are known to reflect changes in the autophagic flux. No significant differences between the two groups of aged mice were detected when ATG7, ATG5, ATG5‐12 and LC3B1 and 2 were measured, suggesting that autophagy was not involved in the biological effect on tau. By contrast, we discovered that brains of HF diet offspring had a significant reduction of pro‐caspase‐3 and cleaved caspase‐3 levels when compared with controls. Interestingly, no differences were noted when we assessed another caspase, caspase‐7.

Previous studies have shown that with aging levels of caspase‐3 in the brain tend to increase and this fact may result in the progressive accumulation of different proteins that are targeted by this protease, among which is tau (Yu et al., 2017a). Based on our results and this information, we believe that the long‐term effect of in utero exposure to HF diet in the offspring is mediated by a reduction of caspase‐3 activation with aging, which resulted in lower accumulation of pathological tau in the brains. This fact was then responsible for better synaptic integrity, which represents the biological substrate for the better performance in the learning and memory tests we observed.

In summary, our paper demonstrates that in utero exposure to a HF diet influences brain health of the offspring later in life by decreasing pathological tau accumulation, improving synaptic integrity, and ameliorating long‐term memory.

Our findings provide new insights on some of the mechanisms that play a functional role in determining whether our brain will be resistant or susceptible to develop a neurodegenerative phenotype later in life. They implicate that research effort on preventing pathological brain aging, which represents the best chance to halt or delay the onset and prevalence of neurodegenerative diseases, as well as the design of dietary interventional approaches, should focus on the early stages of life.

## 4 EXPERIMENTAL PROCEDURES

### 4.1 Animals

All the procedures were approved by the Animal Care and Usage Committee in accordance with the National Institutes of Health guidelines. B6129SF2/J wild‐type mice were used in this study. Mice were kept in a pathogen‐free environment on a 12‐hr light/dark cycle and had access to food and water ad libitum. Ten dams were used for breeding with one male per dam in each cage. After pregnancy was assessed by examining vaginal plug, dams were randomized to receive regular chow (PicoLab 5053: 13% calories from fat, 0.05 calories from cholesterol) (5 dams per strain), or high‐fat diet (Harlan TD88137: 42% calories from fat, 0.2% calories from cholesterol) (5 dams per treatment) throughout the 3 weeks of gestation. After, delivery, all dams were put back on regular chow during lactation. No difference in litter size was observed between diet regimens. No difference in diet compliance was observed between regular chow and high‐fat diet. In average, 4–8 pups per pregnancy were delivered. A total of 8–12 offspring (4–6 males and 4–6 females) were randomized for each experimental condition (WT control: WT CTR; WT high‐fat diet: WT HF). Offspring were kept on regular chow from birth until euthanasia. Offspring from both experimental conditions were assessed for their body weight at 18 months of age.

### 4.2 Glucose tolerance test

Offspring from both experimental conditions underwent glucose tolerance test at 18 months of age. The evening before the test, offspring were housed in a new cage with paper bedding. Food was removed from 5:00 p.m. of the evening before until 9:00 a.m. of the test day. During this time, mice had access to fresh water at libitum. The test day, animals were placed in a restrainer device and the tail vein was punctured with a 23‐ to 27‐gauge needle. Blood drops were collected onto a glucose testing strip (One Touch Ultra Blue strips) and tested for basal glucose concentration at fasting using a commercially available glucose meter (One Touch Ultra 2). Subsequently, animals were injected 2mg/g body weight glucose in the intraperitoneal cavity. 30 min and 2 hr after the injection, glucose concentration was assessed again as described above. After the procedure, animals were returned to their home cage with regular bedding, fresh chow, and water ad libitum.

### 4.3 Cognitive–behavioral test

Offspring from all experimental condition underwent behavioral testing at 18 months of age. All tests were conducted by an experimenter who was blinded to the treatment. The following behavioral tests were implemented.

#### 4.3.1 Y‐maze

Each mouse was placed in the center of a Y‐shaped plastic maze and allowed to explore freely during a 5‐min session for the assessment of spontaneous alternating behavior. An alternation was defined as three subsequent entries in three different arms (i.e., 1, 2, 3 or 2, 3, 1). The percentage alternation score was calculated as: (Total alternation number/total number of entries 2) × 100.

#### 4.3.2 Fear conditioning

The fear conditioning test was conducted in a chamber equipped with black methacrylate walls, a transparent front door, a speaker, and grid floor. For the training phase, each mouse was placed in the chamber and underwent 3 cycles of 30 s sound/10 s electric shock, within a 6‐min time span. The next day, the mouse spent 5 min in the chamber without getting shocked or hearing the sound (contextual recall). At least two hours later, the animal was kept for 6 min in the same chamber but with different flooring, walls, smell, and lighting and heard the cued sound for 30 s (cued recall). During each phase, the freezing activity of the mouse was recorded.

#### 4.3.3 Morris water Maze

The Morris water maze (MWM) test was conducted in a white circular plastic tank filled with opaque water. Mice were trained to find a submerged Plexiglas platform starting from the four cardinal points, every day for a total of 4 days. The fifth day, mice were tested in the probe trial upon reaching the training criterion of 20 s (escape latency). The probe consists of a free 60 s swim in the pool without platform, to assess the number of entries in the zone of the platform and other parameters of interest.

### 4.4 Protein samples preparation

After behavioral tests, mice were euthanized and brains immediately harvested after intraventricular perfusion with PBS buffer, EDTA, protease, and phosphatase inhibitor cocktail. Brains were immediately dissected in two halves: one for biochemistry and the other for immunohistochemistry. For whole protein lysate preparation, 20–40 mg of frozen brain tissue was cut and sonicated for 20 s in 200 to 400 μL of Radioimmunoprecipitation assay buffer (RIPA buffer). Lysates were centrifuged in Beckman Coulter Optima MAX Ultracentrifuge at 100,000 g for 45 min at 4 ͦC. Supernatant was used for Western blot or ELISA analysis of RIPA‐soluble protein fraction. Pellets were resuspended in 30–60 µL of 70% formic acid and neutralized in 6N NaOH to analyze RIPA‐insoluble (formic acid soluble) protein fraction by Western blot and ELISA. Protein lysates were assessed for protein concentration with BCA Protein Assay Kit (Pierce).

### 4.5 RNA samples preparation

Aliquots (20 mg) of frozen brain cortex tissue were sonicated for 20 s in 700 μL of Qiazol reagent (Qiagen). RNA was isolated utilizing miRNeasy isolation kit (Qiagen) according to manufacturer protocol. RNA concentration was assessed utilizing Nanodrop system.

### 4.6 Western blot analyses

RIPA‐soluble protein lysates were separated on sodium dodecyl sulfate (SDS)‐PAGE by using a 10% Bis‐Tris gel and then transferred onto nitrocellulose membranes (Bio‐Rad). Membranes were blocked with Odyssey blocking buffer for 1 hr at room temperature and incubated with primary antibodies overnight at 4°C. After three washing cycles in T‐TBS, membranes were incubated with IRDye 800CW labeled secondary antibody (LI‐COR Bioscience) for 1 hr at room temperature and developed with Odyssey Infrared Imaging System (LI‐COR Bioscience). Primary antibodies used are summarized in Table 1.

**Table 1:** Antibodies used in the study

Antibody	Cat #	Company	MW (kDa)	Dilution	Application
Anti‐Tau	05‐348	Millipore	50	1:200	WB, IHC
Anti‐cleaved tau(Asp421)	AHB0061	TFS	50	1:200	WB, IHC
Anti‐AT180	P10636	TFS	50	1:200	WB, IHC
Anti‐AT8	MN1020	TFS	50	1:200	WB, IHC
Anti‐PHF13	9632	CST	50	1:200	WB, IHC
Anti‐PHF1	sc‐515013	SCB	50	1:200	WB, IHC
Anti‐MC1	gift	Dr. Davies	50	1:200	WB, IHC
Anti‐caspase‐3	sc‐7272	SCB	32/17	1:200	WB
Anti‐caspase‐7	sc‐56063	SCB	35/20	1:200	WB
Anti‐ATG5‐12	ABC14	Millipore	56/33	1:200	WB
Anti‐ATG7	2631	CST	77	1:200	WB
Anti‐LC3B	2775	CST	17/14	1:200	WB
Anti‐SYP	sc‐17750	SCB	38	1:500	WB, IHC
Anti‐PSD95	MA1‐045	TFS	95	1:200	WB, IHC
Anti‐GFAP	sc‐33673	SCB	50	1:200	WB, IHC
Anti‐IBA1	MABN92	Millipore	17	1:200	WB
Anti‐actin‐beta	sc‐47778	SCB	42	1:1,000	WB

Abbreviations: CST, cell signaling technology; IHC, immunohistochemistry; SCB, Santa Cruz Biotechnology; TFS, Thermo Fisher Scientific; WB = Western blot.

### 4.7 Immunohistochemistry

Serial brain sections were cut throughout each brain and mounted on 3‐aminopropyltriethoxysilane‐coated slides. Sections were deparaffinized, hydrated, rinsed with PBS, and pretreated with citric acid for 5 min for antigen retrieval, then with 3% H_2_O_2_ in methanol for 30 min to eliminate endogenous peroxidase activity in the tissue and with blocking solution (5% normal serum in Tris buffer, pH 7.6). Subsequently, sections were incubated overnight at 4°C with primary antibody. The next morning, sections were incubated with secondary antibody and developed using the avidin–biotin complex method (Vector Laboratories) with 3,3‐diaminobenzidine as chromogen. Primary antibodies used are summarized in Table 1.

### 4.8 mRNA expression analysis

Extracted RNA samples were converted to cDNA utilizing RT2 First Strand Kit (Qiagen) according to manufacturer instructions. Gene expression was measured by SYBR‐8 green qPCR technology using RT2 qPCR primer assay (Qiagen) according to manufacturer specification with Applied Biosystems Step One Plus RT–PCR system. The following commercially available primers were used: mouse mapt cat# PPM24640A (Qiagen).

### 4.9 Statistical analysis

All data are expressed as mean ± *SD*. The two‐tailed Student's *t* test was used to compare up to two groups. Statistical significance was set at p < .05.

## CONFLICT OF INTEREST

None declared.

## AUTHOR CONTRIBUTION

A.DM. and D.P. designed the study. A.DM. performed the experiments. A.DM. and D.P. wrote the manuscript. They discussed the results and seen the final version of the paper before submission.
